# Diversity and composition of macroinvertebrate communities in a rare inland salt marsh

**DOI:** 10.1002/ece3.8222

**Published:** 2021-10-20

**Authors:** Abigail E. Cahill, Christopher J. Breen, Irene Corona‐Avila, Cesar A. Cortes, Rosemary Hernandez, Saige Jost, Breh L. K. Ruger, Rachel M. H. Stander, Bach V. Tran

**Affiliations:** ^1^ Biology Department Albion College Albion Michigan USA

**Keywords:** COI, communities, macroinvertebrates, metabarcoding, salt marsh

## Abstract

Inland salt marshes are rare habitats in the Great Lakes region of North America, formed on salt deposits from the Silurian period. These patchy habitats are abiotically stressful for the freshwater invertebrates that live there, and provide an opportunity to study the relationship between stress and diversity. We used morphological and COI metabarcoding data to assess changes in diversity and composition across both space (a transect from the salt seep to an adjacent freshwater area) and time (three sampling seasons). Richness was significantly lower at the seep site with both datatypes, while metabarcoding data additionally showed reduced richness at the freshwater transect end, consistent with a pattern where intermediate levels of stress show higher diversity. We found complementary, rather than redundant, patterns of community composition using the two datatypes: not all taxa were equally sequenced with the metabarcoding protocol. We identified taxa that are abundant at the salt seep of the marsh, including biting midges (*Culicoides*) and ostracods (*Heterocypris*). We conclude that (as found in other studies) molecular and morphological work should be used in tandem to identify the biodiversity in this rare habitat. Additionally, salinity may be a driver of community membership in this system, though further ecological research is needed to rule out alternate hypotheses.

## Graphical abstract

Inland salt marshes are rare habitats in the Great Lakes region of North America, formed on salt deposits from the Silurian period. We used morphological and COI metabarcoding data to assess changes in diversity and composition across both space (a transect from the salt seep to an adjacent freshwater area) and time (three sampling seasons). Community composition was different with different datatypes. Diversity and richness were lowest at the salt seep regardless of datatype.

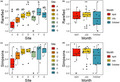

## 1 INTRODUCTION

Abiotic stress can be a determining factor of biodiversity in many communities. Organisms often show trade‐offs between their tolerance to stress, their competitive ability, and their response to disturbance (Grime, 1977; Herbst, 2001; Qi et al., 2018; Sulmon et al., 2015). Habitats that are abiotically stressful are often occupied by fewer kinds of organisms; these species can tolerate the stress and may be excluded via low competitive ability from more benign environments. This general framework of the relationship between biodiversity and stress has been tested empirically (e.g., in coastal salt marshes: Gedan & Bertness, 2009; Kunza & Pennings, 2008) and invoked to explain broad patterns (e.g., the latitudinal or altitudinal species gradient, Rohde, 1992). Abiotic stress is also often invoked in setting range limits on both local and global scales (reviewed in Cahill et al., 2014; Sexton et al., 2009; Sirén & Morelli, 2020; Sunday et al., 2012). However, habitats that are stressful for many organisms often support specialist species that are adapted to local abiotic conditions. Such examples include plants in serpentine soils (reviewed in Brady et al., 2005), the upper intertidal community (Bertness & Ellison, 1987; Stillman & Somero, 1996), or polar habitats (Barnes et al., 2009; Ozheredova et al., 2015).

The intermediate disturbance hypothesis (IDH) predicts that highest diversity will occur in areas with intermediate levels of disturbance: at high levels of disturbance, only the most disturbance‐tolerant species can persist, while at low levels of disturbance, strong biotic interactions dominate and reduce species diversity (Connell, 1978). The IDH model can be expanded to include abiotic stress more generally (e.g., salinity stress), whereby high levels of stress reduce the number of species that can live in an area, and more clement conditions may lead to strong biotic interactions. Although the utility of the IDH as a theoretical framework has been questioned (Fox, 2013), as a verbal model it anticipates that diversity and stress may not be directly, linearly related.

### 1.1 Inland salt marshes

Although salt marshes are generally associated with coastal environments, there are several different types of inland saline habitats (e.g., the Great Salt Lake in the United States, temporary wetlands in the Camargue region of France; Herbst, 2001; Waterkeyn et al., 2008). Inland salt marshes surrounding the Great Lakes region of North America are rare, patchy habitats. They are formed on salt deposits from an ocean that covered the region approximately 400 million years ago, during the Silurian period. In some places, glaciation has removed overlying sediment and groundwater now interacts with the salt (Albert, 2010; Eallonardo & Leopold, 2014; Lincoln et al., 2020). Although never an abundant habitat, inland salt marshes have been very degraded through salt extraction for commercial use, beginning during the settler/colonial period in what is now the United States. Marshes were often chosen as settlement sites due to the role of salt as a critical element in food preservation (Lincoln et al., 2020). We hereafter use “inland salt marshes” to refer exclusively to the habitats that are from this geological origin and which are located generally in the Great Lakes region of North America; we do not further address other types of inland saline habitat.

Inland salt marshes are disconnected from the ocean and exist as patchy habitats in the landscape, designated as critically endangered both on a global scale and within the state of Michigan (Lincoln et al., 2020). The salt constitutes a source of abiotic stress for the species that live there, which are either freshwater species or brackish‐water specialists that have evolved from freshwater ancestors. The plant communities that have been documented at two salt marshes in Michigan and New York, for example, include halophilic species (Eallonardo & Leopod, 2014). Although the plants that have been identified in these marshes are different from those in coastal salt marshes, there is still a clear zonation of plants that can tolerate these salty conditions versus those that cannot (Eallonardo & Leopold, 2014; Lincoln et al., 2020). However, very little is known about the invertebrate species that live in these habitats (Albert, 2010), including basic species composition.

Another potential source of stress in inland salt marshes is the seasonality of the water level. During wetter seasons, the habitat is more suited for animals that require standing water, and in dry seasons, the water in the marsh can dry up altogether. The variability of the water level in ponds and other wetlands has been shown to affect the diversity and composition of plants (Riis & Hawes, 2002) and animals that live in these areas (Anderson & Smith, 2000; Strachan et al., 2014; Uzarski et al., 2004).

### 1.2 Metabarcoding as a technique

One of the problems with characterizing diversity in small invertebrate species (such as those found in wetland sediments) is the difficulty in identifying the species using morphology, which requires a high degree of time and expertise to identify individuals to the species level. Additionally, morphological identification at the species level often cannot be completed for subadults in a population. These problems are not unique to inland salt marsh systems, and DNA‐based species identifications (barcoding) are now routinely used for species identification and monitoring (e.g., Valdez‐Moreno et al., 2012; Viard & Comtet, 2016; Yao et al., 2017). More recently, DNA metabarcoding is also used for these purposes (Afzali et al., 2020; Cahill et al., 2018; Darling et al., 2017; Meyer et al., 2021). Metabarcoding uses high‐throughput DNA sequencing and bioinformatics to identify all members of a community that are present in a sample. Although there are disadvantages to this method (e.g., biases in primers or other steps of the processing protocol, incompleteness of reference databases; Carugati et al., 2015; Danavaro et al., 2016; Cahill et al., 2018), it allows for rapid identification of small organisms, potentially to the species level where such a precise ID is not possible based on morphology (e.g., cryptic species or juvenile life stages; Danavaro et al., 2016; Pearman et al., 2016). The two techniques are complementary rather than redundant: metabarcoding can be inconsistently successful across taxa (Afzali et al., 2020; Cahill et al., 2018; Kelly et al., 2017; Lejzerowicz et al., 2015) while simultaneously allowing more precise and thorough identifications. The combination of morphological and molecular tools is therefore important for verifying the conclusions of a study where possible.

### 1.3 Hypotheses

In this study, we investigated the relationship between abiotic stress (salinity) and diversity in an inland salt marsh. We hypothesized that species diversity and richness would vary with changing salinity in space, though we did not have strong a priori expectations for the shape of the relationship. For instance, diversity/richness might increase with decreasing abiotic stress or might peak at intermediate stress levels. We also measured seasonal change in diversity, richness, and community composition and expected to find temporal changes in these variables. We used both morphological identification and molecular techniques (barcoding and metabarcoding) to address these questions, expecting that metabarcoding would give us the most thorough picture of community composition in the marsh.

## 2 METHODS

### 2.1 Study site

**FIGURE 1:**
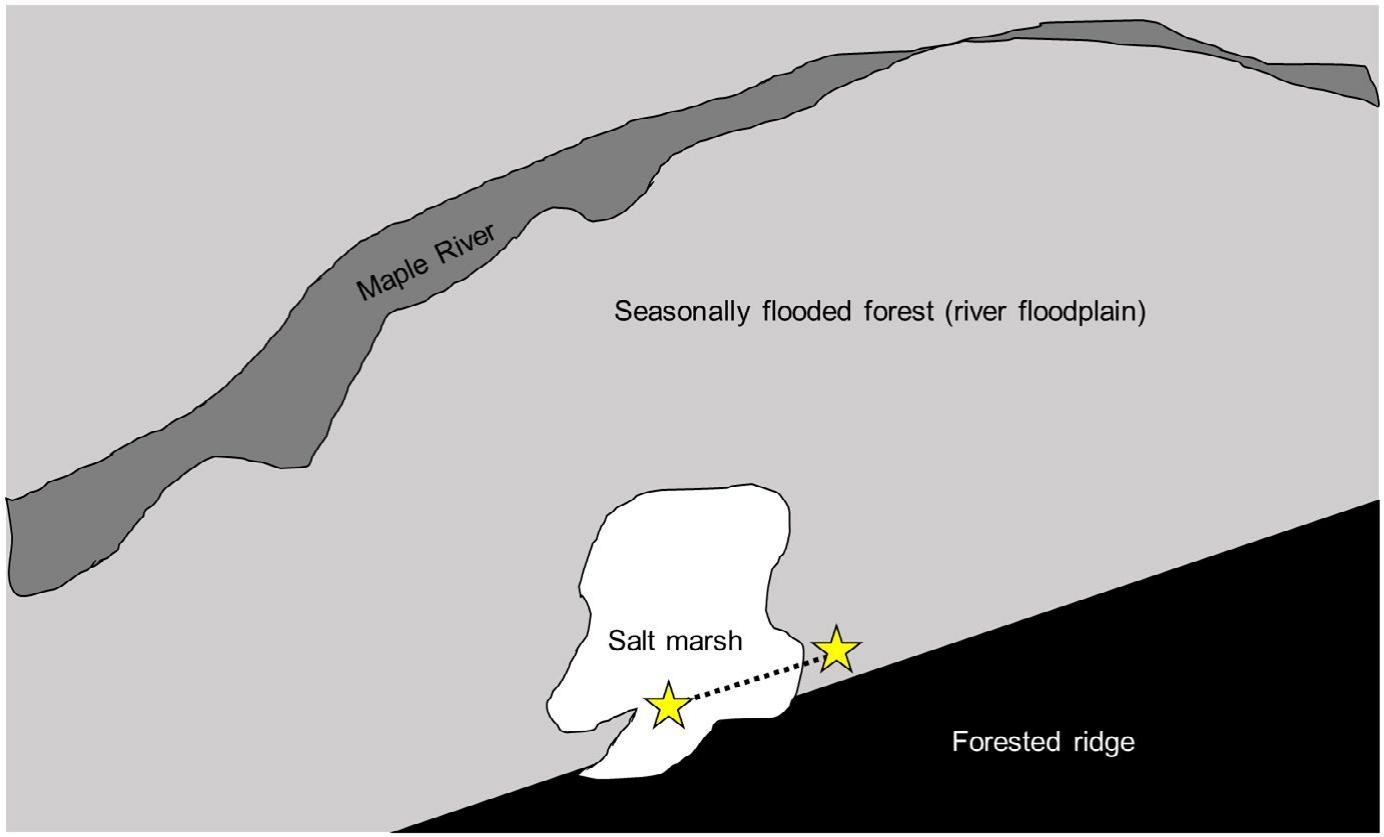
Sampling site in the Maple River salt marsh, after Lincoln et al. (2020). Stars indicate the salt seep (left) and freshwater (right) ends of the transect; the total length of the transect is 120 m, and samples were taken every 20 m. The transect begins at (43.0847N, 84.7649W) and ends at (43.0852N, 84.7642W). White indicates the area affected by the salt seep

The study was conducted in the Maple River inland salt marsh, part of the protected Maple River State Game Area in Fowler, Michigan (USA; 43.0847 N, 84.7649 W). The marsh occupies 6.5 acres, with a small halozone (Lincoln et al., 2020; Figure 1), and the plants present in the area affected by the salt seep have been described by the Michigan Natural Features Inventory (Albert, 2010; Lincoln et al., 2020). Although the seep is within the larger floodplain of the Maple River, it appears to be unaffected by seasonal flooding of the river, with water levels primarily changing due to changes in precipitation and groundwater seepage (Lincoln et al., 2020).

### 2.2 Field sampling

In April, July, and October of 2018, we sampled along a transect within the marsh. The first sample point on the transect was the seep site within the marsh, identifiable because of its lack of vegetation (the rest of the marsh is a dense stand of predominantly *Typha* spp.). We sampled at 7 points along the transect, spaced 20 m apart, for a total transect length of ~120 m. The transect ran parallel to the ridge that forms the boundary of the floodplain (Figure 1). A transect length of 120 m was sufficient to get out of the area influenced by the salt seep: it took us across a small berm, out of the *Typha* marsh, and into an area with different vegetation (including some trees). Three sediment samples were taken from the surface at each transect point, each approximately 450 ml. We used surface sampling because a pilot look at sediment cores showed that invertebrates were only present in the top layer of the marsh. Samples were returned to the laboratory in Albion, Michigan, where half of each sample was preserved at −80°C for molecular analysis and half was preserved in 70% ethanol for morphological identifications.

Soil salinity was measured in the laboratory following the protocol of Rhoades (1996). This involved drying the soil sample, weighing ten grams of each sample, suspending it in 100 ml of water, agitating it every ten minutes for an hour, and then measuring total salinity (in psu) with a YSI probe (model Pro2030). Since the organisms in the sample were living in the porewater of the sediment, soil salinity is an ecologically relevant measure of the environment.

### 2.3 Morphological data collection

Each sediment sample was filtered at 100 µm and rinsed with DI water, then placed in a sorting tray and examined under a dissecting microscope. Animals that were larger than ~1 mm were removed and identified using a dichotomous key (University of Wisconsin Extension, 2014). Identifications were done to the lowest taxonomic level that could be easily and reliably reached; this was often the class or family level but varied among taxonomic groups. The complete list of taxa used in morphological identifications is available in Appendix S1. At least two replicate samples were sorted per transect point, totaling at least 14 samples at each of the three sampling timepoints (44 samples overall).

### 2.4 Analyses: Morphology

We calculated both rarefied taxon richness and Simpson's diversity index for each sample and used two‐way analyses of variance to compare these indices across season and across the transect points (hereafter “sites”). We used permutational analysis of variance (PERMANOVA) and nonmetric multidimensional scaling (NMDS) plots to analyze and compare community composition across space and time. All analyses were conducted in R, version 3.5.3 (R Core Team, 2019), with the package vegan (version 2.5‐5, Oksanen et al., 2019).

### 2.5 COI barcoding of target specimens

Two species of arthropod were consistently found in high population densities at the salt seep: an ostracod and a ceratopogonid midge larva. To get a more precise species identification, we performed COI barcoding. We extracted DNA from a one midge larva and one ostracod using Qiagen DNeasy kits and amplified a ~650‐bp fragment of cytochrome oxidase I using universal LCO and HCO primers (Folmer et al., 1994). PCRs were done with GoTaq® PCR reageants from Promega (Madison, WI, USA), and each reaction contained 4 µl buffer, 2 µl MgCl_2_, 1 ul dNTP, 0.4 µl each of the forward and reverse primers, 0.1 µl Taq polymerase, 8 µl water, and 4 µl of DNA. The thermocycler program was as follows: [95°C × 3 min, 35× (95°C × 40 s, 45°C × 40 s, 72°C × 1 min), 72°C × 3 min]. Sanger sequencing was performed at the Genomics Core of the Research and Technology Support Facility at Michigan State University. Sequences were identified using BLASTn (NCBI).

### 2.6 Metabarcoding

For metabarcoding, we extracted DNA from approximately 10 g of each soil sample using Qiagen DNeasy PowerMax Soil Kit. We used all samples collected or 21 per timepoint (63 total samples). Samples from each timepoint were extracted and sequenced separately (Table 1). We purified extracted genomic DNA using Qiagen DNeasy PowerClean Cleanup Kit. We used universal metazoan primers mlCOIint and HCO2198 from Leray et al. (2013) to amplify a fragment of the COI gene. Each reaction contained 10 µl of Phusion High‐Fidelity Master Mix (Thermo Fisher Scientific), 0.4 µl of each of the forward and reverse primers, 6.7 µl water, and 2.5 µl DNA. The reactions were run with the following protocol: [98°C × 3 min, 27× (98°C × 10 s, 46°C × 30 s, 72°C × 45 s), 72°C × 5 min]. Each sample was analyzed with three replicate PCRs. After verifying the PCRs’ success on 1.5% agarose gels, we pooled the three replicates together (21 total pools per timepoint) and quantified their DNA concentration using a Qubit. Sequencing was performed on a MiSeq v2 Nano flow cell with 2 × 250 bp paired‐end reads at the Genomics Core of the Research Technology Support Facility (RTSF) at Michigan State University. Raw reads were demultiplexed by the RTSF.

**TABLE 1:** Summary of sequencing output

Month	N reads	N OTUs	Mean reads per OTU	N taxonomic categories
April	473,058	8,548	37.04 (± 8.91)	72
July	282,812	7,206	51.69 (± 125.1)	68
October	334,935	7,378	30.81 (± 9.76)	71

Note

For each month (= sequencing run), the number of quality reads retained after bioinformatic processing in MOTHUR, the number of OTUs these reads represent, the mean number of reads per OTU (± *SD*), and the number of taxonomic categories following identification using the BOLD database. The number of taxonomic categories was calculated after the removal of fungi, algae, and unidentifiable OTUs.

### 2.7 Analyses: Metabarcoding

We analyzed the metabarcoding data using MOTHUR version 1.40.5 (Schloss et al., 2009), with the MiSeq standard operating procedure described in Kozich et al. (2013). Sequences from all three sampling timepoints were analyzed in a single run of the bioinformatic pipeline. The paired ends were joined with make.contigs. We used screen.seqs (maxlength = 370, maxhomop = 8, maxambig = 0) for quality filtering and then produced unique sequences using unique.seqs. The reference sequences produced in this step were aligned using align.seqs and a reference from the Barcode of Life Database (BOLD; Ratnasingham & Herbert, 2007). Pre‐clustering was undertaken with pre.cluster (diffs = 3) and chimeras removed using vsearch (Edgar, 2010). Sequences were identified using the classify.seqs command from MOTHUR v.1.39.5 implemented in Galaxy, with the public server at usegalaxy.org (Afgan et al., 2018). We compared our sequences to BOLD for identification.

Using all OTUs (not just ones that were identified), and after removing OTUs with 10 or fewer reads, we conducted a permutational multivariate analysis of variance (PERMANOVA) to analyze community composition across the seven different sites. We also conducted a separate PERMANOVA comparing the three sampling seasons. These PERMANOVAs were followed with nonmetric multidimensional scaling analyses (NMDS) based on Bray–Curtis distances. All data were fourth‐root transformed prior to these analyses. We also calculated both rarefied taxon richness and Simpson's taxon diversity indices for each sample and used two‐way ANOVAs to measure differences among sites on the transect and among seasons. These analyses were performed using the vegan package (version 2.5‐5, Oksanen et al., 2019) in R version 3.5.3 (R Core Team, 2019).

To look at taxon‐specific patterns of diversity and composition, we used the subset of reads that could be identified based on the BOLD database. After removing reads that were not identified or that belonged to non‐animal taxa, we identified the nine taxa with the greatest number of reads both overall and within Insecta. We then repeated the analyses (PERMANOVA, NMDS, diversity/richness calculations) with only the OTUs that were identified as insects.

### 2.8 Analyses: Comparisons

In order to compare the results from the two different datatypes, we correlated the diversity indices and rarefied richness values that were calculated in each sample with the two types of data. Since not all samples were analyzed for morphology, only those with both metabarcoding and morphological data were included here (*N* = 44 samples). In order to compare community composition, we compiled a matrix of Bray–Curtis distances for each site pair with each datatype and used a Mantel test to compare the distance matrices between datatypes. These analyses were conducted for each of the three sampling dates combined. For all of these comparative analyses, the morphological dataset was compared with the full metabarcoding dataset with all OTUs.

## 3 RESULTS

### 3.1 Salinity data

**FIGURE 2:**
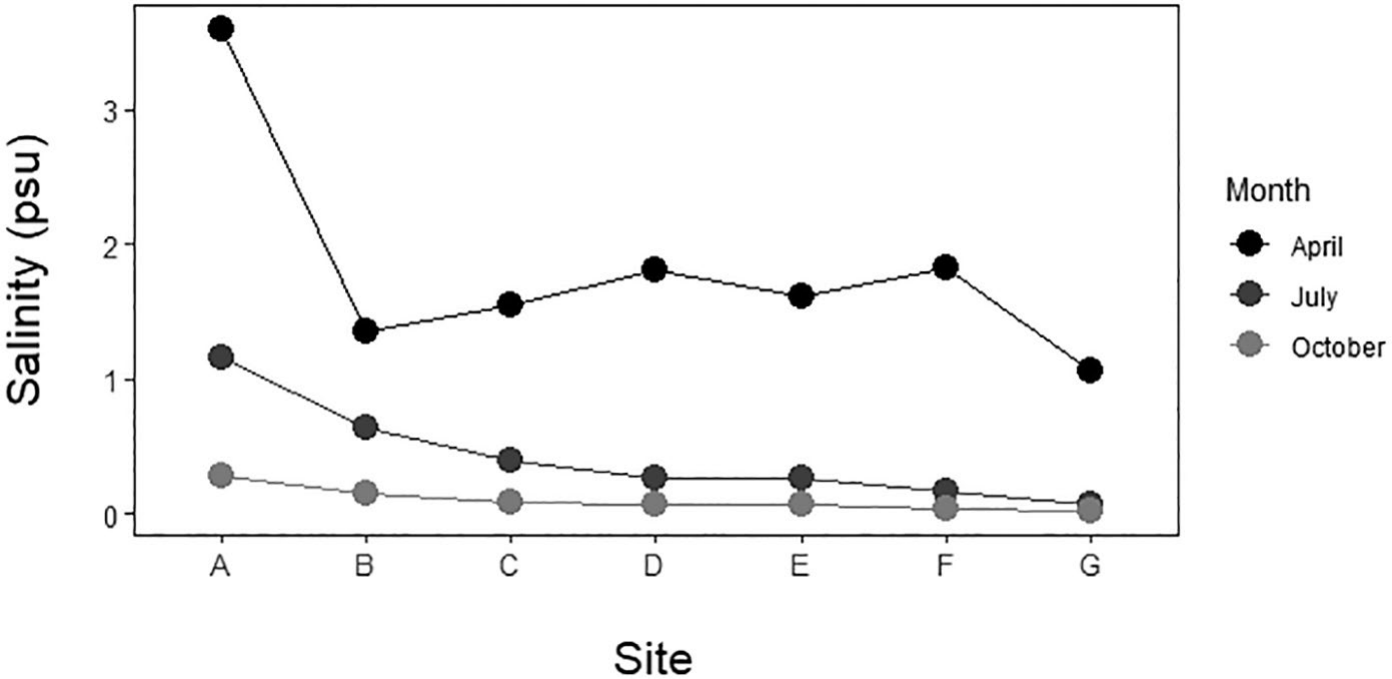
Salinity changes in space and time. The salt seep corresponds to point A, the freshwater end of the transect to point G, and all points were sampled 20 m apart

The salinity in the marsh ranged from 0.025 to 3.597 psu, depending on the time of year and point on the transect. Mean salinity in April was 1.83 psu, while in July it was 0.43 psu and 0.10 psu in October. The first point, located in the seep, was consistently the saltiest, and salinity dropped rapidly moving away from the seep (Figure 2; Appendix S5).

### 3.2 Morphology

**FIGURE 3:**
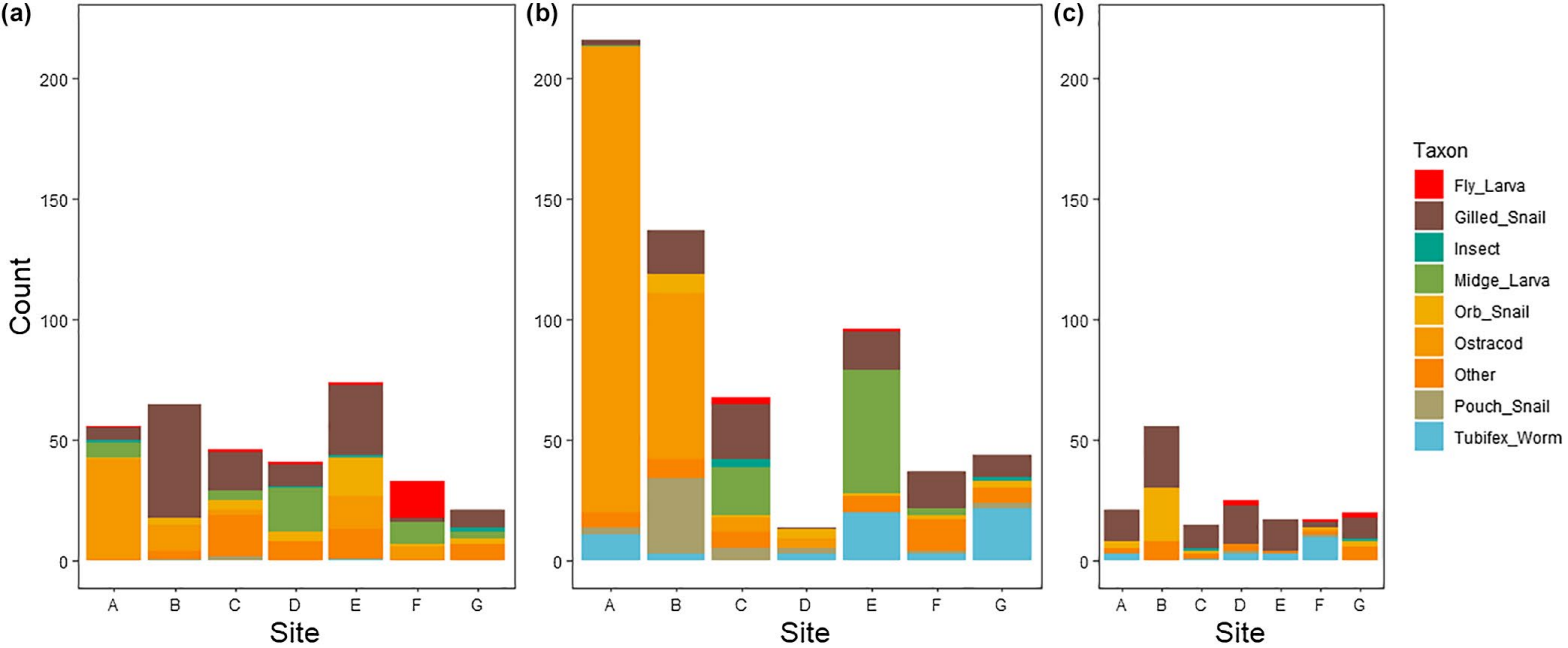
Composition plot of the nine most commonly identified taxonomic groups in April (panel a), July (panel b), and October (panel c), based on morphological data. Site A corresponds to the sampling point at the salt seep, and site G is the sampling point at the opposite end of the transect

We found 25 different taxa using morphology‐based identifications. The most widely distributed taxon was gilled snails (Gastropoda: prosobranch pond snails), which appeared in nearly every sample, but the most abundant taxon in terms of overall numbers was an ostracod (possibly *Heterocypris salina*, see barcoding results below), which were found primarily at the seep site itself (total number found = 346; Figure 3). Community composition clearly shifted over the course of the year (Figure 3). Ostracods were most abundant in July, while the October samples were dominated by gilled snails. Worms were more abundant in July and October than in April. The complete morphological dataset is found in Appendix S1.

**FIGURE 4:**
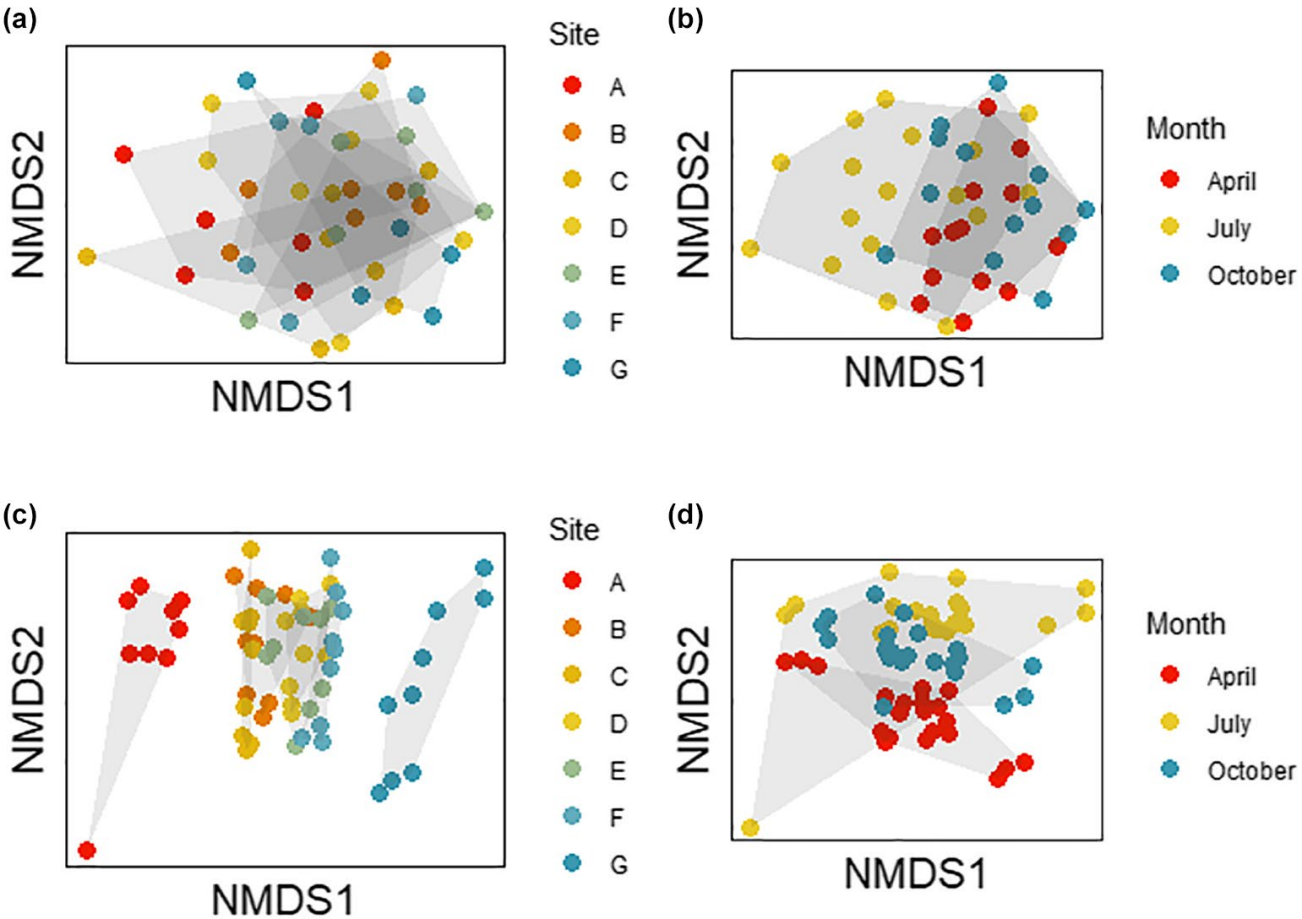
Nonmetric multidimensional scaling plot based on morphological data (a and b) and molecular data (c and d), with points coded according to site along the transect (panels a and c, where A corresponds to the saltwater seep and G to the freshwater end) and season (panels b and d). The stress in panels a and b is 0.244; the stress in panels c and d is 0.181

Whole‐community analyses (PERMANOVA) revealed significant effects of both transect point and season, as well as their interaction, on community composition (Table 2). The NMDS analysis shows an overlap of sampling points, but clearer separation by season (Figure 4): April and October overlap in composition but July has a larger variance. This was due to a larger number of terrestrial animals that were present in July (when there was much less water in the marsh), including ticks, spiders, collembolans, and earthworms.

**TABLE 2:** Community composition

Source of Variation	*df*	MS	pseudo‐*F*	*p*
Morphological data				
**Site**	**6**	**0.519**	**2.616**	**<.001**
**Season**	**2**	**0.786**	**3.959**	**<.001**
Site*Season	**24**	**0.328**	**1.650**	.**004**
Error	44	0.199		
Molecular data				
**Site**	**6**	**0.653**	**1.579**	**<.001**
**Season**	**2**	**0.823**	**1.989**	**<.001**
Site*Season	**12**	**0.487**	**1.177**	**<.001**
Error	42	0.414		
Molecular data—insects only				
**Site**	**6**	**0.379**	**3.871**	**<.001**
**Season**	**2**	**0.381**	**3.813**	**<.001**
Site*Season	12	0.112	1.146	.131
Error	42	0.098		

Note

PERMANOVA results of morphological (top), molecular (middle; all OTUs), and insect‐only (bottom) data. Analyses compared points on the transect (site) and sampling time (season) in a two‐way PERMANOVA. Molecular data were fourth‐root transformed prior to analysis. Significant effects at *p* < .05 are highlighted in bold.

**FIGURE 5:**
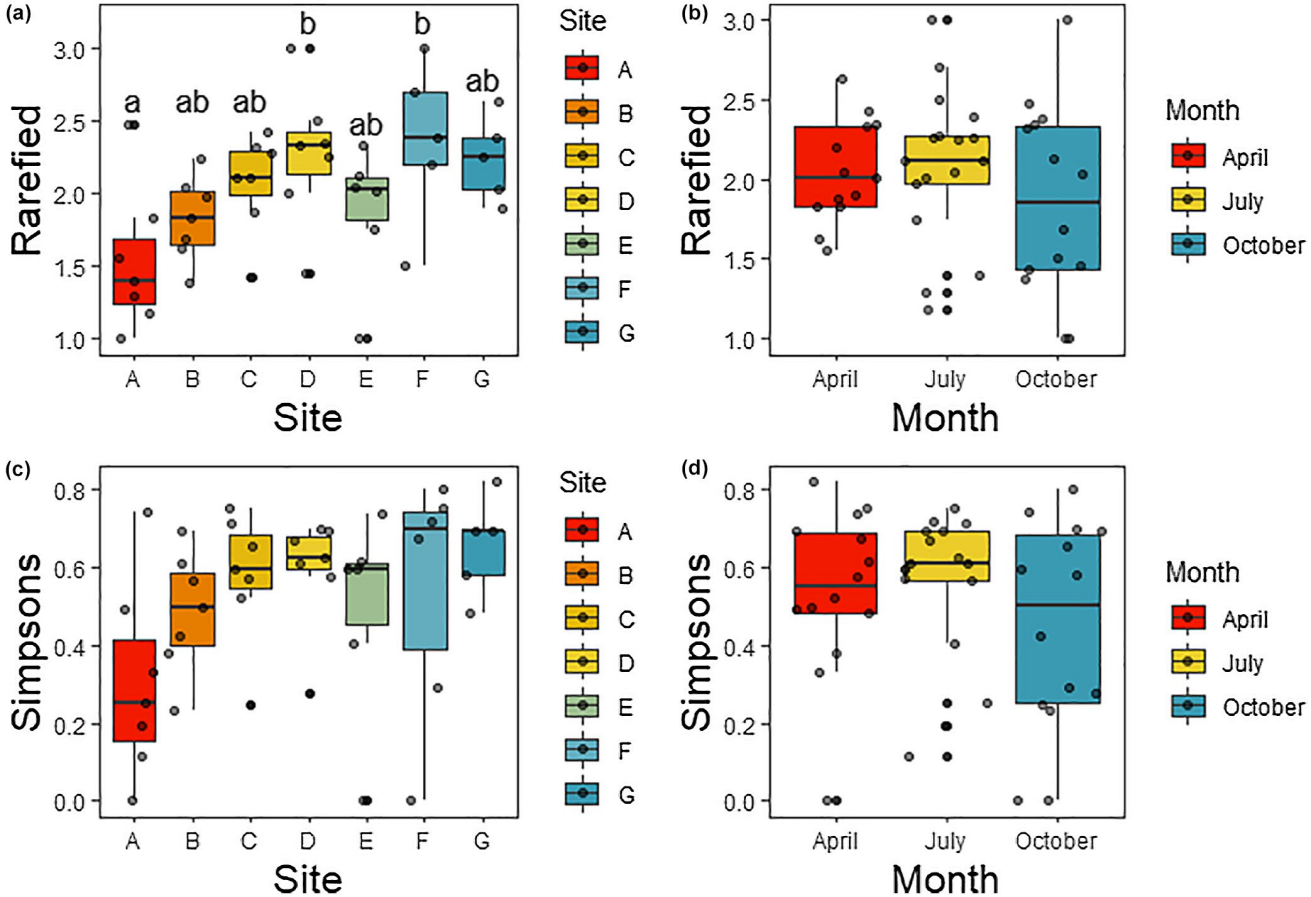
Taxon richness (rarefied richness, panels a, b) and diversity (Simpson's Index, panels c, d) based on morphological data. Panels a & c present the data grouped by site (where A is the saltwater seep and G is the freshwater end of the transect), and panels b & d present the data grouped by sampling time. Letters in panel a represent significant differences according to Tukey's HSD post hoc tests

There was no effect of season on the rarefied taxon richness (*p* = .385), but there was a significant effect of site (*p* = .035). The seep site had lower richness than two points in the center of the transect based on Tukey's HSD post hoc tests (Figure 5). There was no interaction between site and season (*p* = .856; Table 3, Figure 5). There was no significant effect of site, season, or their interaction on Simpson's index of taxonomic diversity (Table 3), though there was a visible trend toward lower diversity at the seep site (Figure 5). Rarefaction curves are available in Appendix S4: Figures S1–S4.

**TABLE 3:** Richness and diversity

Source of variation	Rarefied taxonomic richness				Simpson's Index of taxonomic diversity			
	*df*	MS	*F*	*p*	*df*	MS	*F*	*p*
Morphological data								
Site	**6**	**0.577**	**2.779**	.**035**	6	0.086	1.752	.152
Season	2	0.207	0.996	.385	2	0.050	1.028	.373
Site*Season	12	0.115	0.554	.856	12	0.037	0.757	.685
Error	23	0.208			24	0.049		
Molecular data								
Site	**6**	**78782**	**12.31**	**<.001**	6	7.9 e−05	1.470	.212
Season	2	15934	2.49	.095	2	5.7 e−05	1.052	.358
Site*Season	12	7926	1.52	.155	12	6.8 e−04	1.057	.419
Error	42	6399			42	5.4 e−05		
Molecular data—insects only								
Site	6	0.094	1.392	.240	**6**	**0.032**	**2.906**	.**018**
Season	2	0.018	0.263	.770	2	0.011	0.962	.391
Site*Season	12	0.096	1.416	.197	12	0.016	1.477	.172
Error	42	0.068			42	0.011		

Note

ANOVA of richness (left) and diversity (right) metrics among different transect points (site) and sampling times (seasons). Top: morphological identifications. Middle: All molecular operational taxonomic units (OTUs) were considered. Bottom: Only OTUs from class Insecta were considered. Significant effects at *p* < .05 are highlighted in bold.

### 3.3 COI barcoding

The COI barcoding of the midge larva resulted in only 302 bp of high‐quality DNA. This relatively short fragment yielded equal identity matches in BLAST to two species of biting midge (Diptera: Ceratopogonidae): 99.7% to both *Culicoides sonorensis* and *C*. *variipennis*. The ostracod sample resulted in a sequence of 555 bp of high‐quality DNA with a BLAST match to *Heterocypris salina* (Ostracoda: Cyprididae), with a 95.9% identity score. However, between a small fragment size in the midge larva and a relatively low percent identity match in the ostracod, any attempt to identify the individuals to species based on these barcodes is premature. The sequences have been submitted to GenBank, with the accession numbers MZ444699 and MZ444700.

### 3.4 Metabarcoding results and community analysis

The bioinformatic pipeline recovered 16 617 unique OTUs from 1 090 805 quality‐filtered reads. We conducted composition and diversity analyses on this full dataset as described above. The full set of OTUs and their abundances is available in Appendix S2; the list of identified OTUs and abundances is in Appendix S3. There was variation in the numbers of reads, OTUs, and identified OTUs recovered among months (Table 1, Table S1).

The PERMANOVAs conducted with all OTUs showed that sites were strongly different across the transect (Table 2). The NMDS plot revealed that the two ends of the transect (salt and freshwater) are clearly different, with the intermediate points clustering in the center of the plot (Figure 4). There was also differentiation according to season (Table 2), and the NMDS plot showed that July again had a wider variation than April or October, driven by a point at the seep site in July where over 99% of sequences were from a single OTU (later identified as a mite in the order Sarcoptiformes). The interaction of site and season was also significant in the PERMANOVA (Table 2).

**FIGURE 6:**
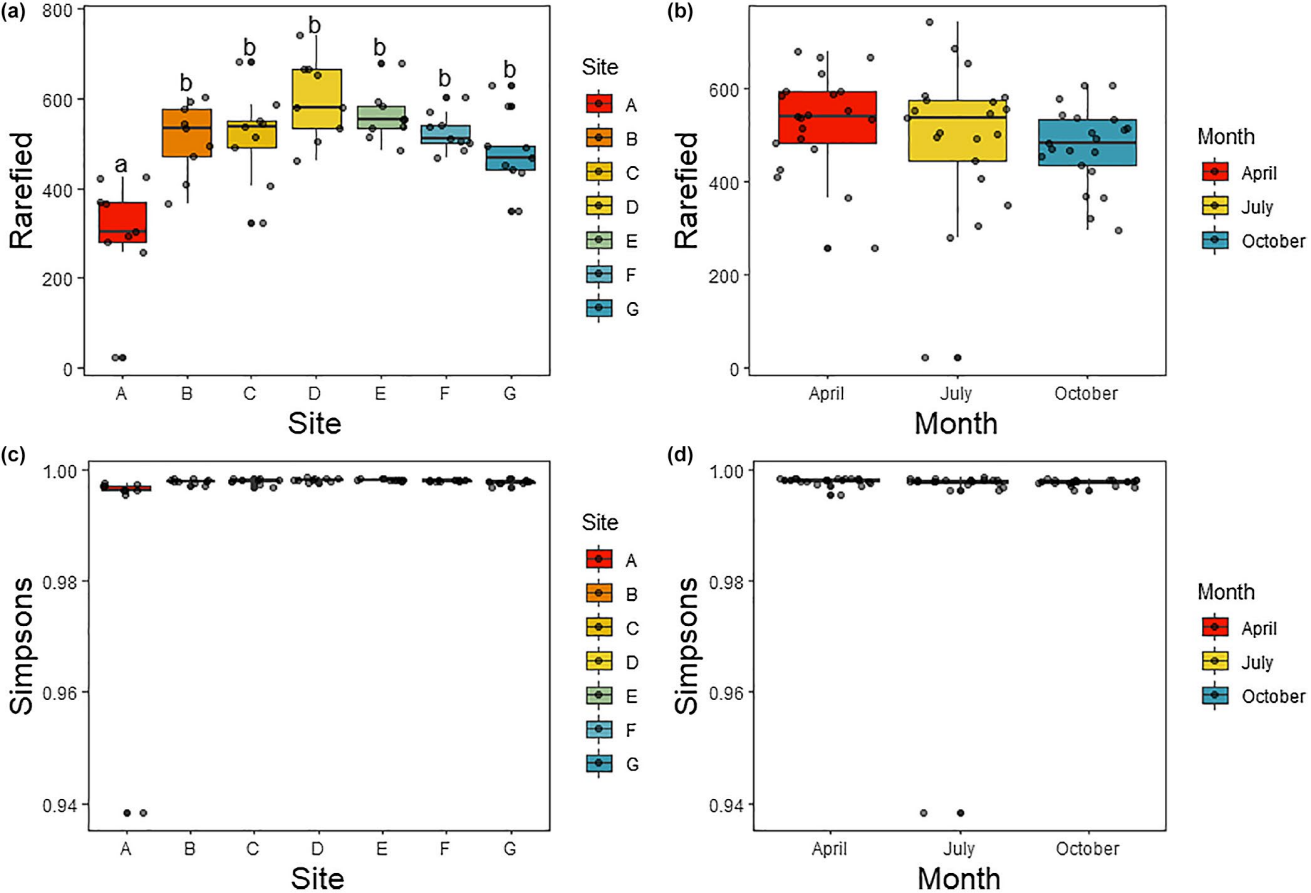
Taxon richness (rarefied richness, panels a, b) and diversity (Simpson's Index, panels c, d) based on metabarcoding data (all OTUs considered). Panels a & c present the data grouped by site (where A is the saltwater seep and G is the freshwater end of the transect), and panels b & d present the data grouped by sampling time. Letters in panel a represent significant differences according to Tukey's HSD post hoc tests

Based on all OTUs, there was a significant effect of sampling site on rarefied taxon richness (*p* < .001), but not of season (*p* = .095) nor of the interaction between site and season (*p* = .155; Table 3). Richness showed a relationship where the seep had lower richness than all other sites (Tukey's HSD tests; Figure 6). Although not statistically significant after corrections for multiple testing, there is a hump‐shaped relationship in richness (with richness highest in the center of the transect; Figure 5). Taxon diversity (Simpson's index) was not different based on site or season, nor was there an interaction between the two (Table 3, Figure 6).

The PERMANOVA of only the OTUs identified as Insecta showed a significant effect of both site and season on insect community composition, though no interaction between the two (Table 2). As with the full OTU dataset, there was a slight difference between the seep community and other sampling points, but the difference among replicates was much less striking than with the full OTU dataset (Figure S5). In contrast with the full OTU dataset, there was no difference in rarefied richness according to either sampling site or season (Table 3). Simpson's diversity was not different among seasons, but was lower at the seep than in the center of the transect (Figure S6).

### 3.5 Taxon identifications based on metabarcoding data

**FIGURE 7:**
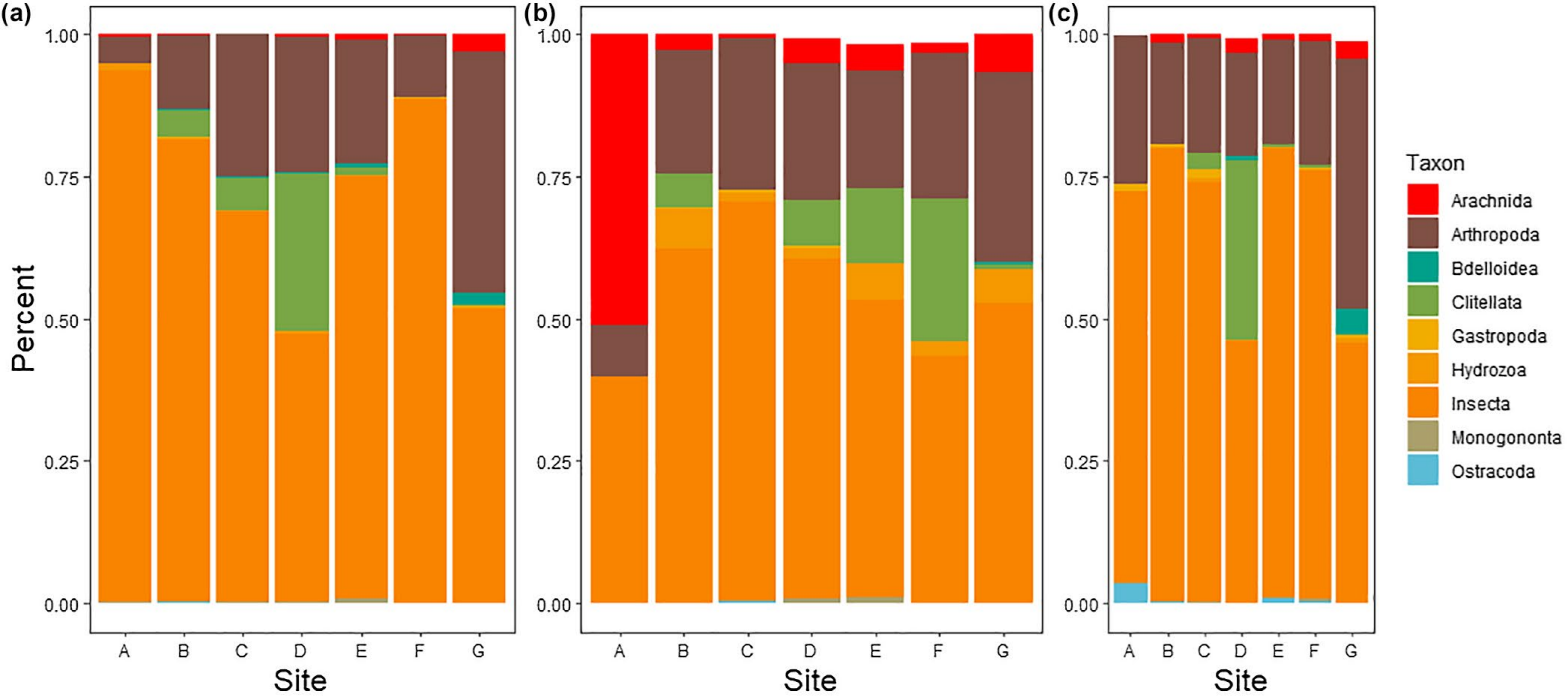
Composition plot of the most commonly identified taxonomic classes in April (panel a), July (panel b), and October (panel c), based on metabarcoding data. Values correspond to the percentage of reads represented by each taxonomic group within each site. Site A corresponds to the sampling point at the salt seep, and site G is the sampling point at the opposite end of the transect

In addition to whole‐community analyses, we found the animal taxa that were most commonly represented in the dataset. For these analyses, we removed OTUs that were identified as fungus (8954 reads) or algae (26,651 reads), as well as the 54% of reads which were unable to be classified using the BOLD database. This left 108 taxonomic groups from 462,308 reads. Taxonomic identifications ranged from the phylum level (e.g., “Unidentified Arthropoda”) to the species level. The most abundant OTUs in all samples were from arthropods, particularly insects (Figure 7, Appendix S3). The most abundant taxonomic classes identified by the analysis pipeline, in decreasing order of abundance, were Insecta (arthropods), unclassified Arthropoda, Clitellata (annelids), Arachnida (arthropods), Hydrozoa (cnidarians), Bdelloidea (rotifers), Gastropoda (molluscs), Monogononta (rotifers), and Ostracoda (arthropods). We identified a few interesting patterns in the data: annelid worms were most abundant toward the center of the transect with a peak that shifted through the seasons (probably in response to changing water levels), while ostracods were patchily abundant (Figure 6). We found many taxa with the metabarcoding that were not identified with morphological techniques; conversely, gastropods, which were commonly found in microscope samples, were relatively rare in the metabarcoding samples (Figure 7).

**FIGURE 8:**
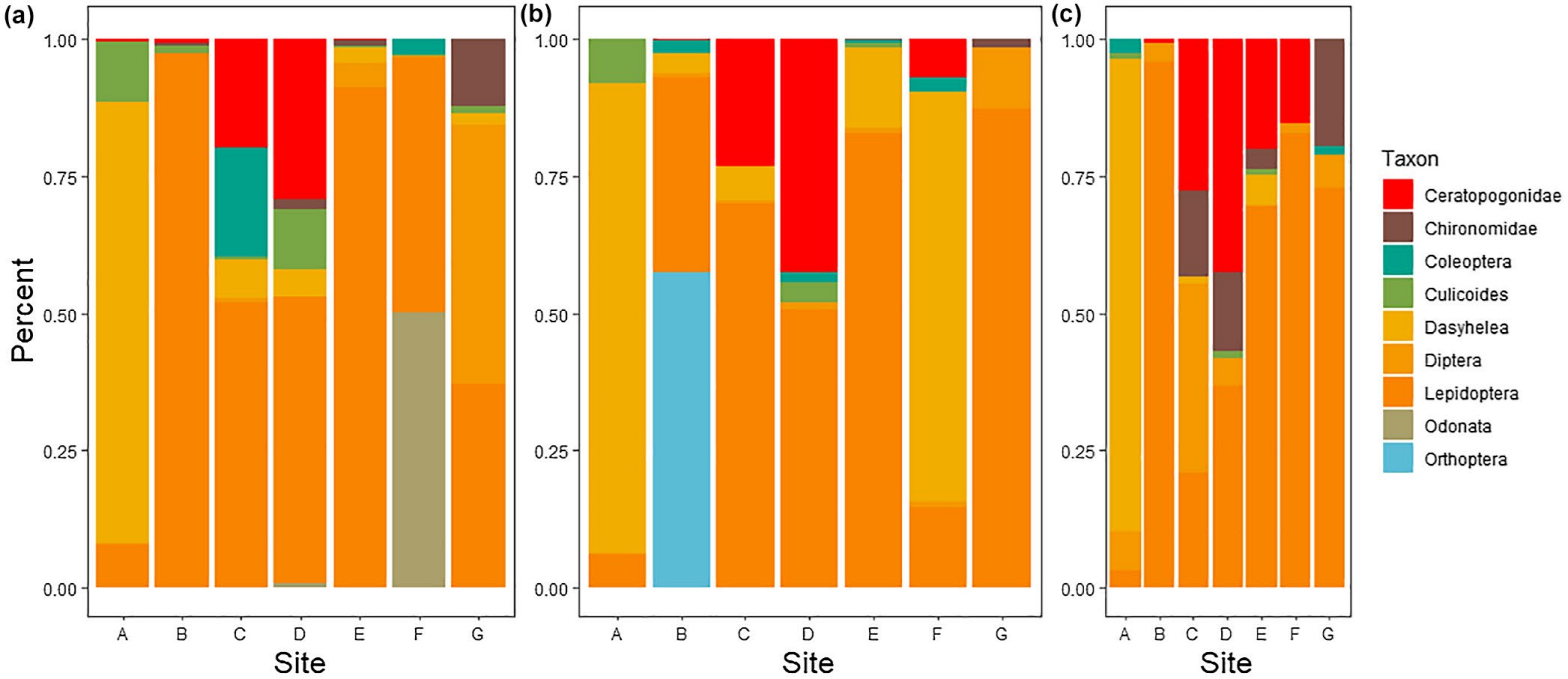
Composition plot of the most commonly identified taxa within Insecta (after the removal of unclassified insect sequences) in April (panel a), July (panel b), and October (panel c), based on metabarcoding data. Values correspond to the percentage of reads represented by each taxonomic group within each site. Site A corresponds to the sampling point at the salt seep, and site G is the sampling point at the opposite end of the transect

When we looked at community composition within the insect sequences (and after removing sequences that could not be identified lower than Insecta), the most common taxa in descending order were Lepidoptera spp., *Dasyhelea* sp. (Diptera: Ceratopogonidae), Odonata spp., Ceratopogonidae spp. (Diptera), Diptera spp., Coleoptera spp., Orthoptera spp., *Culicoides* sp. (Diptera: Ceratopogonidae), and Chironomidae spp. (Diptera) (Appendix S3, Figure 8). It is notable that three of the most common taxa are found in the dipteran family Ceratopogonidae (the biting midges) and that other dipteran groups are also present in large numbers. However, the total number of reads from all dipteran groups combined was still far less than the number of lepidopteran reads in the dataset. *Dasyhelea* and *Culicoides* reads were concentrated at the seep site itself, with other ceratopogonid midges in the center of the transect and lepidopteran reads relatively evenly spaced (though rare at the seep site; Figure 8).

### 3.6 Comparison of methods

Mantel tests revealed a weak relationship between the two datasets (metabarcoding and morphology) when all OTUs were considered and when data were separated based on sampling site (Mantel's *r* = .438, *p* = .07; Table 4), but no relationship between methods when data were separated based on season (Mantel's *r* = −1, *p* = 1; Table 4).

**TABLE 4:** Comparison of community composition

Among sites							
	A (salt seep)	B	C	D	E	F	G
A (salt seep)		*0.401*	*0.354*	*0.334*	*0.356*	*0.367*	*0.462*
B	0.875		*0.365*	*0.496*	*0.459*	*0.547*	*0.553*
C	0.840	0.751		*0.366*	*0.318*	*0.375*	*0.417*
D	0.904	0.766	0.782		*0.198*	*0.256*	*0.374*
E	0.898	0.751	0.781	0.730		*0.298*	*0.413*
F	0.924	0.800	0.817	0.747	0.743		*0.421*
G (freshwater)	0.973	0.921	0.930	0.890	0.896	0.875	

Among seasons			
	April	July	October
April		*0.333*	*0.364*
July	0.806		*0.425*
October	0.749	0.709	

Note

Community dissimilarities among sites (top panel) and sampling seasons (bottom panel) based on both morphological (above‐diagonal elements, italics) and molecular data (below‐diagonal elements). Numbers are Bray–Curtis measures of community dissimilarities, where values closer to 1 represent higher amounts of dissimilarity between communities.

**FIGURE 9:**
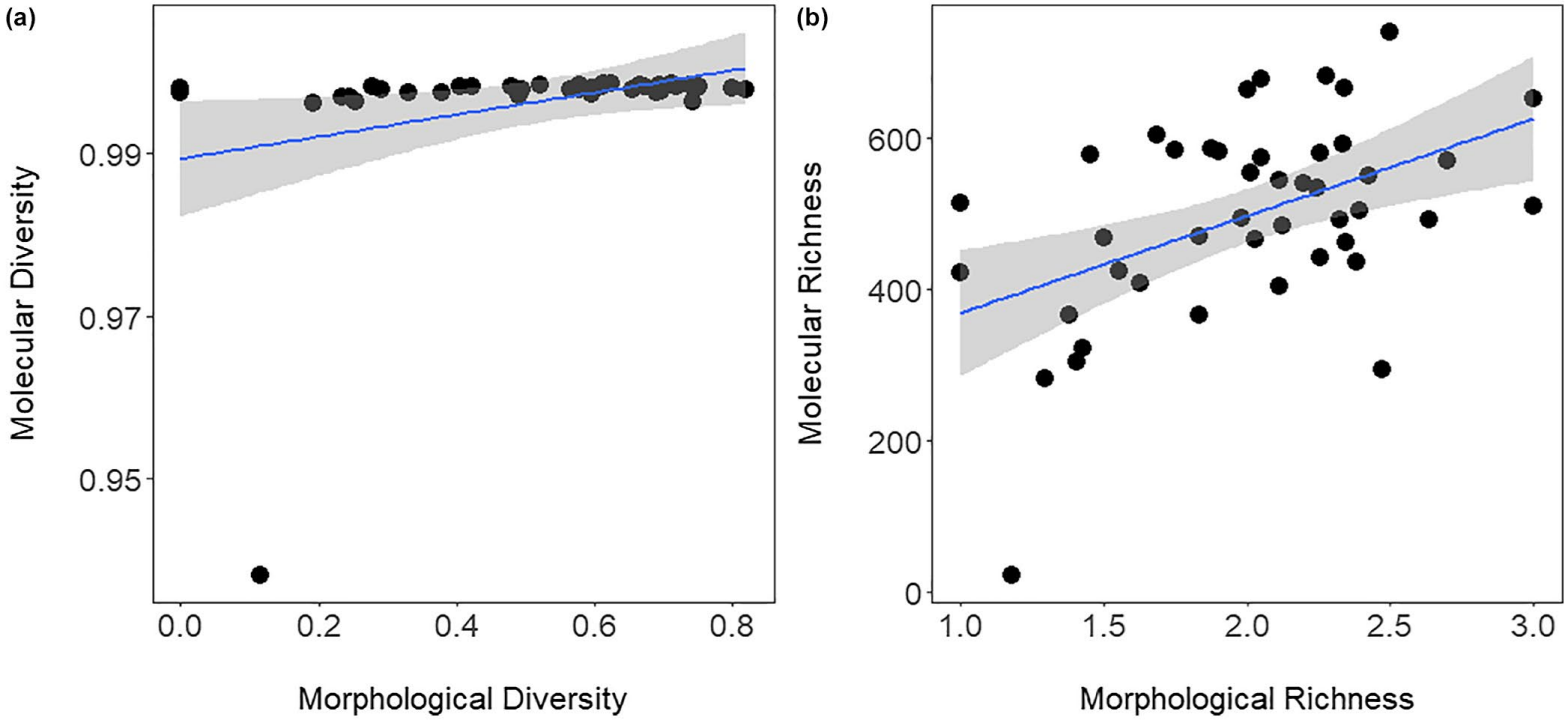
Correlation of morphological and molecular diversity (Simpson's Index, panel a) and richness (rarefied richness, panel b)

There was a weak but significant correlation between Simpson's diversity metrics calculated with morphological and molecular data (all OTUs; *r* = .321, *p* = .033; Figure 9). There was also a correlation in rarefied richness between the two methods (*r* = .473, *p* = .001; Figure 9).

## 4 DISCUSSION

Our study describes the invertebrate community composition in a rare inland salt marsh over both a spatial gradient and a temporal one, corresponding with salinity stress. We found that both spatial and temporal changes are drivers of the community composition and diversity, but that the details depend on the analysis and datatype considered. As many other studies have found, the information gained by using morphological and molecular data together is complementary, though both richness and diversity were correlated between datasets.

Both community composition and species richness changed with sampling site using the metabarcoding data. The NMDS analysis showed a distinct community at the seep, a very different community at the freshwater end of the transect, and a group of sites in the middle that were not clearly differentiated from each other. Looking more closely at the OTU data revealed that the center of the transect contained both groups that were abundant in the more freshwater sites (e.g., earthworms) and those that were abundant closer to the seep (e.g., ostracods, ceratopogonid midges), and this contributed to high richness values in the transect center. Conversely, NMDS analysis showed strong overlap between the three seasons, but this was mainly due to one outlying point in the July samples. At this point, from the seep site, over 99% of reads were identified as an unclassified species of Sarcoptiformes (a mite). It seems likely that this is due to one large individual that dominated the PCR and sequencing; this in turn decreased diversity and richness metrics at that point.

The relatively low taxonomic precision of the morphological dataset precluded statistically significant patterns of composition, and NMDS did not reveal differentiated communities along the transect. However, richness increased with distance from the seep site, and diversity showed a weak trend in this direction as well. Like the metabarcoding data, there was no effect of season on diversity or richness. The NMDS analysis of the morphological data again showed that July was much more variable in terms of community composition; April and October strongly overlapped with each other.

The data recovered with traditional morphological identifications are of a much coarser scale than the molecular data and did not give clear results on how community composition changes. Additionally, in large part because of how the sediment was processed for these morphological identifications (e.g., selecting animals >1 mm for identification), many taxa could not have been identified. For example, two of the most common taxa in the molecular data were Bdelloidea and Monogononta, classes of Rotifers. These organisms are smaller than our morphological size limit, transparent, and can survive complete desiccation (Caprioli et al., 2004), making it exceedingly unlikely that our morphological IDs would have seen them.

None of these weaknesses of morphological data are unique to our study, and all have been discussed at length elsewhere (e.g., Cahill et al., 2018; Danavaro et al., 2016; Kelly et al., 2017). However, without the morphological dataset, we would have missed key patterns in the marsh. Most notably, some of the most common invertebrates in the morphological dataset were several groups of gastropods. However, gastropods were much rarer in the metabarcoding dataset (though still in the list of top‐ten taxa with the most reads). This result is consistent with other papers, which have found that molluscan DNA is less prevalent in metabarcoding data, including with the primers that we used (Beentjes et al., 2019; Cahill et al., 2018; Kelly et al., 2017). This may relate to the difficulty of crushing the shells of microgastropods during the lysis steps of the DNA extraction process, meaning that the starting DNA pool may contain less molluscan DNA relative to the more easily digested arthropods and annelids. Additionally, the primers we used (Leray et al., 2013) are putatively universal, but seem to work better with arthropods and annelids than molluscs (Cahill et al., 2018).

Another surprising result in the metabarcoding data was the very high number of reads from lepidopterans (butterflies and moths); this was the most abundant order of insects. Lepidopteran OTUs were present in all replicates, usually with a particular OTU being present in only a single replicate. Despite this, we did not see a single lepidopteran larva or adult in the morphological analysis. One possible explanation here is puddling: a behavior that butterflies exhibit in which they dabble in puddles to increase their salt intake. Male butterflies in particular need salt for proper gamete formation (Sculley & Boggs, 1996). Through puddling, butterflies would be expected to leave eDNA that could be collected in our samples. Another possible explanation for the large number of lepidopteran reads is birds that prey on the insects and then excrete butterfly DNA; this seems like a less likely explanation because we had no identified reads from Aves in the dataset (Appendix S3). We also note that other technical sources of error (e.g., primer bias) might be better explanations, as eDNA seems unlikely to overwhelm the results in this way. It is also possible that we did not identify lepidopterans in any of the morphological samples purely by chance and that they are in fact present in the marsh sediments.

Both datasets revealed a large number of ceratopogonid midges (biting midges). DNA barcoding identified the species at the seep itself as either *Culicoides sonorensis* or *C*. *variipennis*, and metabarcoding identified four OTUs belonging to this family, in at least two genera. Both *C*. *sonorensis* and *C*. *variipennis* are found in brackish and polluted habitats (e.g., coastal salt marshes and liquid manure pools in agricultural facilities; Schmidtmann et al., 2000), so their presence in the Maple River marsh is unsurprising. *Culicoides sonorensis* is a vector of bluetongue virus, a livestock disease (Maclachlan & Mayo, 2013; Purse et al., 2005). The barcoded individual was a very close match to both species. *Culicoides variipennis* and *C*. *sonorensis* are part of a species complex (Holbrook et al., 2000) and were until recently described as two subspecies of *C*. *variipennis*. Although *Culicoides* sp. were found primarily at the seep site based on the morphological data, ceratopogonid sequences were identified along the transect. Further work, both based on barcoding and on morphological identification, is underway to identify the particular species found at the seep, as well as some of the ceratopogonids identified in the metabarcoding data.

We conducted these analyses using sites as a rough proxy for salinity, given their increasing distance from the salt seep. However, as Figure 2 shows, salinity varied dramatically particularly at the seep site during the year, and there was also seasonal variation in the relationship between site and salinity. When we regressed rarefied taxon richness against salinity, rather than site, there was no relationship in either the morphological dataset (*p* = .524) or the metabarcoding dataset (*p* = .370). There were also no significant relationships between salinity and taxon diversity in either the morphological dataset (*p* = .549) or the OTU dataset (*p* = .598). Given these results, and the lack of seasonal signal in community composition based on our NMDS plots, we therefore conclude that although taxa seem to respond to salinity stress in the long‐term (i.e., by the development of groups of salt‐ and fresh‐associated taxa), short‐term (seasonal) variations in salinity do not strongly reshuffle the community in the marsh, even over the short spatial scale of our transect. Some taxa do show seasonal shifts in their abundance along the transect—this is particularly evident in annelid worms in both the morphological and molecular analyses, which are found in the center of the transect in April and October and move toward the seep during our July sampling. However, the worms may be responding to water level rather than salinity per se; there was no standing water at the seep in July and we saw an incursion of terrestrial fauna at that time (e.g., arachnids, beetles). In fact, a major limitation of our study is that we did not systematically examine other ecological variables, such as water depth, organic content of the sediment, or vegetation cover, and so we cannot include them into our quantitative analyses. Although water depth was spatially and seasonally variable, along the transect it was consistently shallow, never exceeding 1 m during our sampling period. Emergent vegetation is nonexistent at the seep itself, but points farther from the seep are in a *Typha*‐dominated marsh (Lincoln et al., 2020), which should also influence the invertebrate community (though we note that the difference in vegetation across the marsh is a response to salinity; Albert, 2010; Lincoln et al., 2020).

Another possible driver of composition in the marsh is dispersal from the Maple River itself. Although we cannot rule out this idea, other sampling we have done in the floodplain near the edge of the river shows that this community is full of isopods, damselfly larvae, and other species more characteristic of flowing water (data not shown). Additionally, all of our sampling sites are roughly equidistant from the river (Figure 1), meaning that the strong spatial differences we find within the marsh are not the result of only distance to the river. In future work, we hope to explore other potential drivers of these invertebrate communities, including the relative roles of community assembly versus species sorting in this habitat.

## 5 CONCLUSIONS

Our results demonstrate that the biodiversity and composition of invertebrate fauna in this inland salt marsh depend on distance from the salt seep, which may be related to abiotic salinity stress. Diversity and richness were lower at the seep site, and metabarcoding data supported a hump‐shaped relationship where intermediate levels of salinity allow the coexistence of taxa from both ends of the transect. Similar to other studies, we found that molecular data provide higher precision but that not all taxa were sampled equally well, making the continued use of both datatypes essential in this system. We also identified a few taxa that are common at the relatively high‐salinity seep, and work is underway to investigate the salt tolerance and adaptation of these species, as well as the role of other ecological factors in determining diversity patterns in space and time in this rare habitat.

## CONFLICTS OF INTERESTS

The authors have no conflicts of interest.

## AUTHOR CONTRIBUTION

**Abigail E. Cahill:** Conceptualization (lead); Data curation (lead); Formal analysis (lead); Funding acquisition (lead); Investigation (lead); Methodology (lead); Project administration (lead); Resources (lead); Software (lead); Supervision (lead); Validation (lead); Visualization (lead); Writing‐original draft (lead); Writing‐review & editing (lead). **Christopher J. Breen:** Data curation (supporting); Investigation (supporting); Writing‐review & editing (supporting). **Irene Corona‐Avila:** Data curation (supporting); Writing‐review & editing (supporting). **Cesar A. Cortes:** Data curation (supporting); Investigation (supporting); Writing‐review & editing (supporting). **Rosemary Hernandez:** Data curation (supporting); Investigation (supporting); Writing‐review & editing (supporting). **Saige Jost:** Data curation (supporting); Investigation (supporting); Writing‐review & editing (supporting). **Breh L. K. Ruger:** Data curation (supporting); Investigation (supporting); Writing‐review & editing (supporting). **Rachel M. H. Stander:** Data curation (supporting); Investigation (supporting); Writing‐review & editing (supporting). **Bach Tran:** Data curation (supporting); Investigation (supporting); Writing‐review & editing (supporting).

## Supporting information

Appendix S1

Appendix S2

Appendix S3

Appendix S4

Appendix S5

## Data Availability

Raw sequence reads have been deposited at the NCBI Sequence Read Archive with the project number PRJNA741798; COI sequences for the ostracod and midge larva have been deposited in GenBank with accession numbers MZ444699 and MZ444700, respectively. The datasets of OTUs and taxon identification (morphological and molecular) are available as supplementary materials and as a Dryad repository, https://doi.org/10.5061/dryad.bg79cnpbv.
