# Supplementary material for: Diversity and composition of macroinvertebrate communities in a rare inland salt marsh
Source: Ecol Evol. 2021 Oct 20;11(21):14351–65. doi: 10.1002/ece3.8222 (PMC8571600; doi:10.1002/ece3.8222)
Supplement: Supplementary file 4 — Appendix S4 [file ECE3-11-14351-s002.docx]

**Appendix 4: Supplemental Figures and Tables**


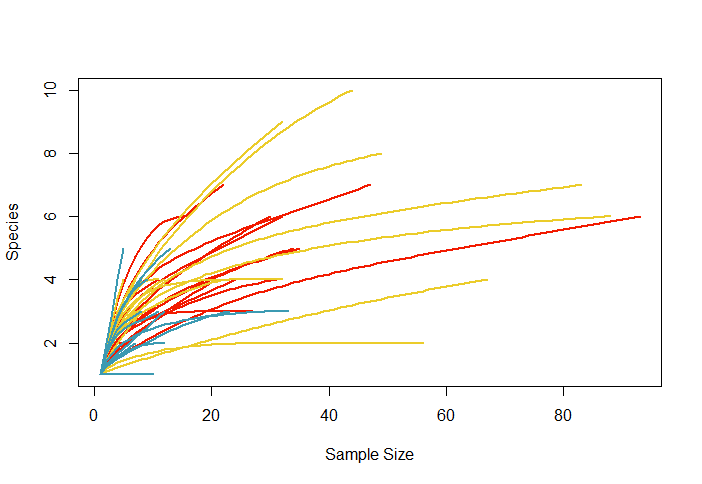


**Figure S1:** Rarefaction curve for the morphological data from each sample, colored by month (red = April, yellow = July, blue = October).


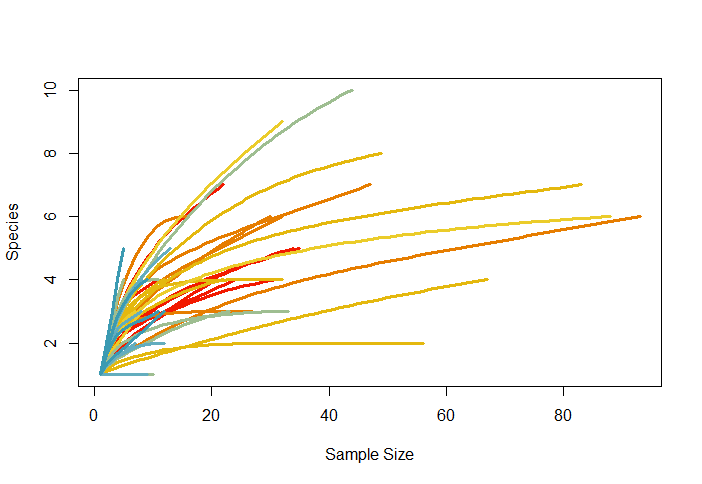


**Figure S2:** Rarefaction curve for the morphological data from each sample, color coded by site (red = seep, blue = fresh; intermediate sites are represented by a gradient of colors moving from warm or closer to the seep to cool or farther from the seep).


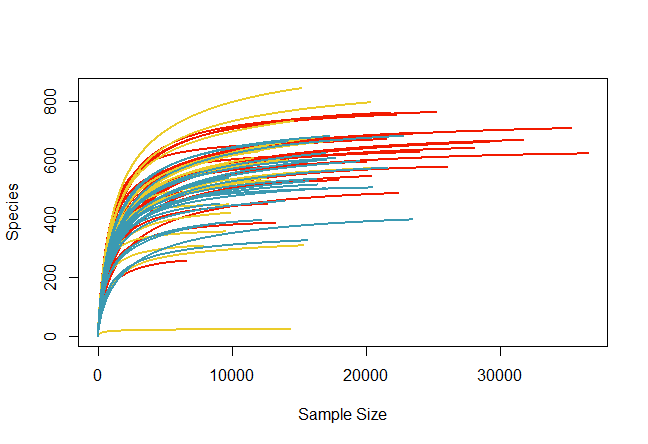


**Figure S3:** Rarefaction curve for the molecular data from each sample, colored by month (red = April, yellow = July, blue = October). Sample size = number of reads.


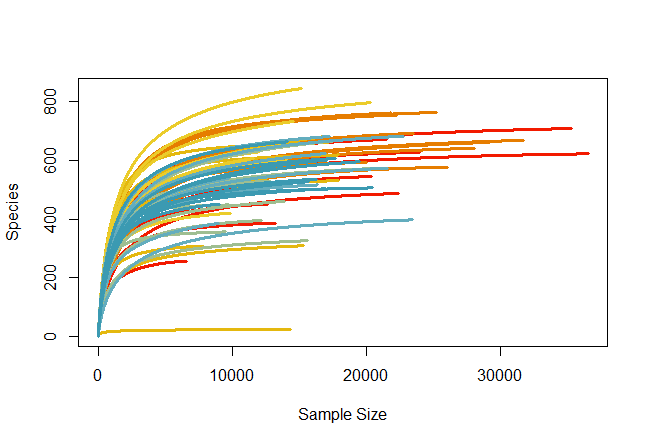


**Figure S4:** Rarefaction curve for the molecular data from each sample, color coded by site (red = seep, blue = fresh; intermediate sites are represented by a gradient of colors moving from warm or closer to the seep to cool or farther from the seep). Sample size = number of reads.


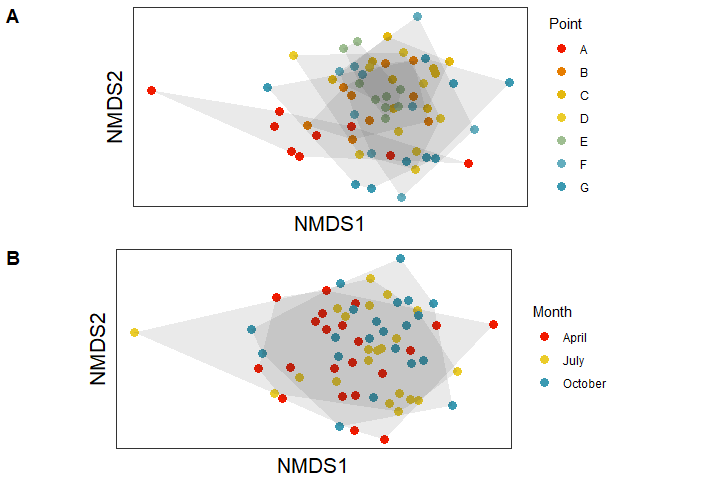


**Figure S5:** NMDS plots of insect OTUs, separated by site (A) and season (B). Stress = 0.263.


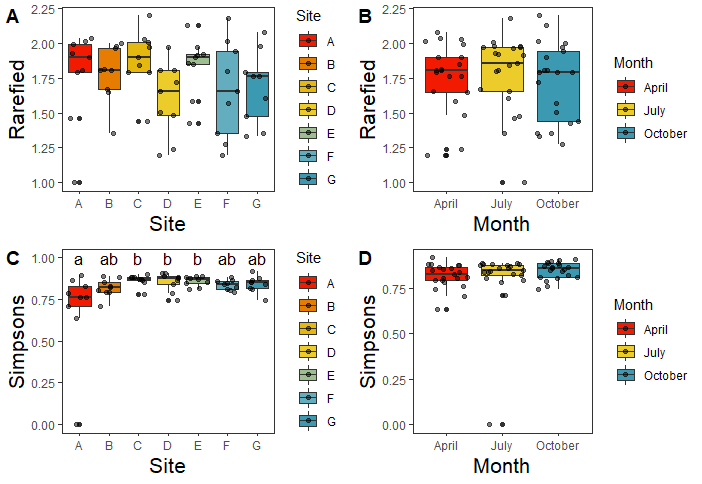


**Figure S6:** Rarefied richness (panels A and B) and Simpson’s diversity (panels C and D) plots of insect OTU data. Data are coded by both site (A & C) and season (B & D). Letters in panel C represent significant differences at p = 0.05.

**Table S1:** Summary of sequencing output for all 63 replicates.

| Month | Point | Number of reads | Mean reads per OTU |
| --- | --- | --- | --- |
| April | A | 6577 | 25.59144 |
| April | A | 12646 | 28.03991 |
| April | A | 13224 | 34.25907 |
| April | B | 21519 | 32.07004 |
| April | B | 20385 | 37.40367 |
| April | B | 22351 | 45.89528 |
| April | C | 35316 | 49.88136 |
| April | C | 36518 | 58.71061 |
| April | C | 23912 | 38.13716 |
| April | D | 29142 | 44.08775 |
| April | D | 22230 | 29.48276 |
| April | D | 21765 | 28.60053 |
| April | E | 23533 | 34.00723 |
| April | E | 19983 | 33.69815 |
| April | E | 25184 | 33.00655 |
| April | F | 28025 | 43.72075 |
| April | F | 26043 | 45.13518 |
| April | F | 31672 | 47.41317 |
| April | G | 17943 | 33.79096 |
| April | G | 17901 | 28.96602 |
| April | G | 17189 | 26.04394 |
| July | A | 7842 | 25.46104 |
| July | A | 14326 | 596.91667 |
| July | A | 15318 | 49.57282 |
| July | B | 16809 | 24.71912 |
| July | B | 8687 | 15.5681 |
| July | B | 12510 | 23.20965 |
| July | C | 9823 | 23.3881 |
| July | C | 20265 | 25.45854 |
| July | C | 14101 | 23.58027 |
| July | D | 15119 | 17.91351 |
| July | D | 12038 | 19.13831 |
| July | D | 14542 | 19.86612 |
| July | E | 11550 | 19.05941 |
| July | E | 13723 | 21.11231 |
| July | E | 13514 | 22.4485 |
| July | F | 21134 | 36.88307 |
| July | F | 18184 | 27.18087 |
| July | F | 12832 | 24.25709 |
| July | G | 9736 | 21.73214 |
| July | G | 11264 | 21.45524 |
| July | G | 9495 | 26.67135 |
| October | A | 15607 | 47.87423 |
| October | A | 13863 | 30.13696 |
| October | A | 12141 | 30.73671 |
| October | B | 8933 | 23.2026 |
| October | B | 16922 | 27.16212 |
| October | B | 17233 | 25.30543 |
| October | C | 21547 | 37.60384 |
| October | C | 23390 | 58.91688 |
| October | C | 9827 | 17.30106 |
| October | D | 16326 | 31.63953 |
| October | D | 22775 | 33.39443 |
| October | D | 9715 | 18.02412 |
| October | E | 16270 | 30.35448 |
| October | E | 17031 | 28.33777 |
| October | E | 17659 | 29.14026 |
| October | F | 15685 | 29.76281 |
| October | F | 19471 | 32.72437 |
| October | F | 14119 | 21.29563 |
| October | G | 20476 | 40.4664 |
| October | G | 9001 | 19.95787 |
| October | G | 16944 | 33.68588 |

**Table S2:** PERMANOVA results of insect OTU data. Significant results at p = 0.05 are indicated in **bold**.

| Source of Variation | df | MS | pseudo-F | p |
| --- | --- | --- | --- | --- |
| **Site** | **6** | **0.379** | **3.871** | **< 0.001** |
| **Season** | **2** | **0.373** | **3.813** | **< 0.001** |
| Site*Season | 12 | 0.112 | 1.146 | 0.131 |
| Error | 42 | 0.098 |  |  |
